# Editorial: Vibratory therapy: impacts on endocrine disorders

**DOI:** 10.3389/fendo.2026.1878558

**Published:** 2026-06-01

**Authors:** Mario Bernardo-Filho, Francois Constant Boyer, Redha Taiar, Danúbia da Cunha de Sá-Caputo

**Affiliations:** 1 Laboratório de Vibrações Mecânicas e Práticas Integrativas, Departamento de Biofísica e Biometria, Instituto de Biologia Roberto Alcantara Gomes and Policlínica Universitária Piquet Carneiro, Universidade do Estado do Rio de Janeiro, Rio de Janeiro, Brazil; 2 Instituto Saúde.com ltda, Rio de Janeiro, RJ, Brazil; 3 University of Reims Champagne-Ardennes, Equipe Associée de Recherche 3797, Reims, France; 4 Physical and Rehabilitation Medicine Department, Sebastopol Hospital, 48 rue de Sébastopol, Reims, France; 5 Université de Reims Champagne-Ardenne, MATériaux et Ingénierie Mécanique (MATIM), Reims, France

**Keywords:** endocrine system, hormone, mechanical vibration, systemic vibratory therapy, vibratory therapy

## Introduction

Vibratory therapy (VT) is an intervention in which the human body is exposed to mechanical vibration (MV). It is a potential therapeutic or adjunct training approach for various health conditions. The presence of mechanoreceptors at the superficial and internal levels of the human body highlights the relevance of MV to the promotion of health. Systemic vibratory therapy (SVT) involves whole-body vibration (WBV) exercises, which are produced when MV generated by vibrating platforms is transmitted throughout the body (1).

**Figure 1:**
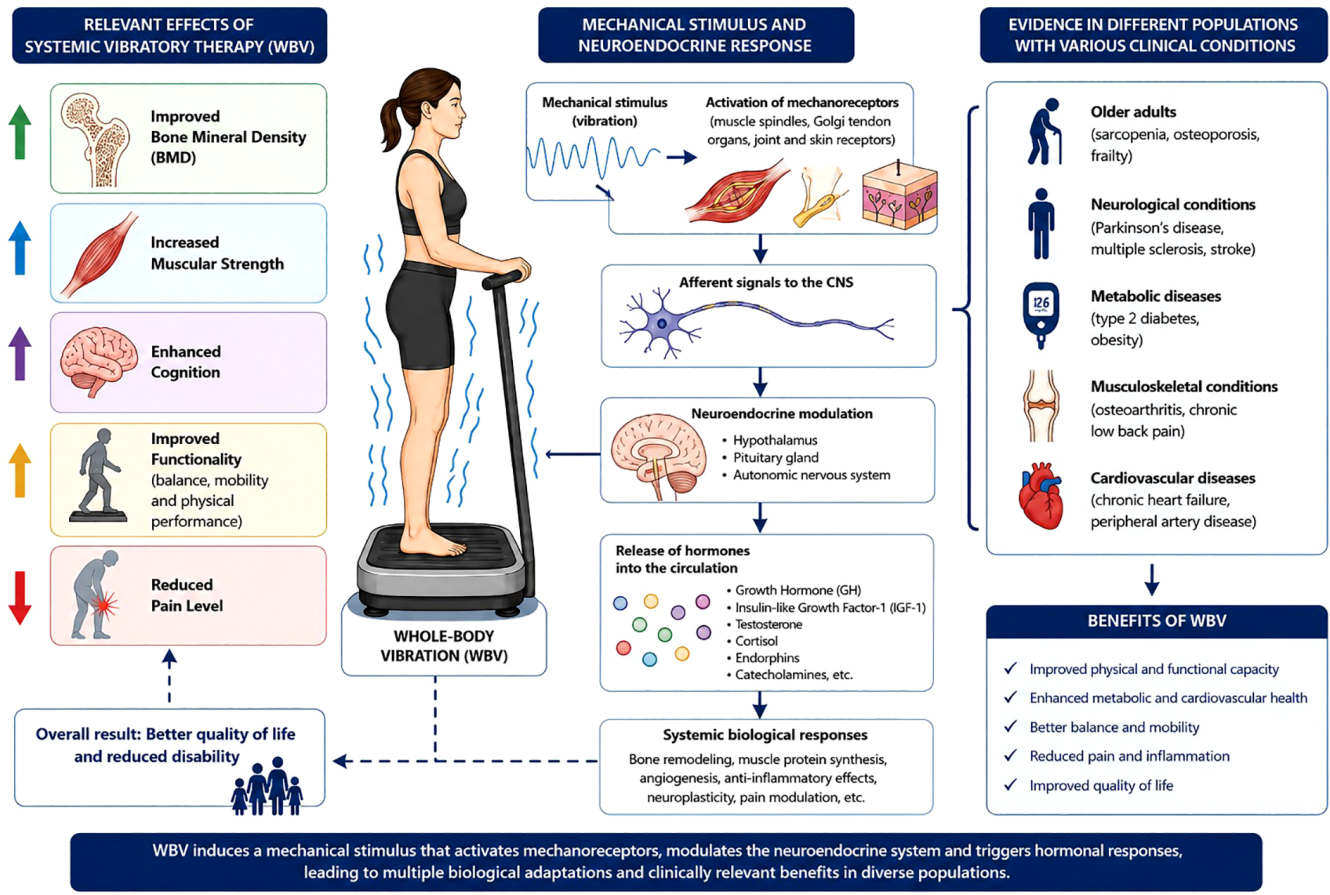
biological system influenced by WBV intervention. This figure was generated by artificial intelligence (ChatGPT).

The Relevant effects of SVT include improvements in bone mineral density (BMD) (2), muscular strength (3), cognitive function (4), functionality, and reduced pain level (5). The action of the mechanical stimulus in mechanoreceptors may favor the release of various hormones into the bloodstream by endocrine glands (**Figure 1**). Several biological responses related to the neuroendocrine system have been reported in different populations with various clinical conditions and benefits of the WBV exercises have been demonstrated (6, 7).

Further mechanistic studies on muscle dynamics demonstrate that vibration stimulation increases muscle contractile force and promotes bone formation through mechanical loading. Furthermore, clinical trials demonstrate the potential of VT in improving lumbar and hip bone density, enhancing bone biomechanical properties, and reducing fracture risk. These effects are particularly notable when VT is combined with drugs such as bisphosphonates or teriparatide, as they exhibit synergistic effects.

SVT is used in various clinical applications, but its relevance for managing endocrine disorders has not been extensively investigated. SVT’s intriguing potential in hormone regulation and metabolic enhancement is seen as an innovative adjunct approach to treating conditions such as osteoporosis, obesity and diabetes; however, scientific evidence remains sparse.

VT therapy is increasingly investigated as a complementary non-pharmacological intervention in osteoporosis, obesity, sarcopenia, and metabolic diseases. However, current evidence suggests that passive vibratory stimulation alone is unlikely to constitute the optimal therapeutic stimulus but rather as a mechanobiological stimulus whose biological and clinical effects are likely enhanced when combined with active muscle contractions and structured physical exercise. From a mechanistic perspective, VT may be interpreted within the broader framework of mechanotransduction. The 2021 Nobel Prize in Physiology or Medicine, awarded to 8, highlighted the fundamental importance of mechanosensitive receptors and ion channels in converting physical stimuli into biological signals.

In this Research Topic, ideas, approaches, opinions, and comments regarding the latest research providing evidence on the impact of WBV intervention on biological systems are shared. In fact, the main aim of this Research Topic is to share scientific information about the role of VT in certain diseases related to the endocrine system, such as osteoporosis, obesity and diabetes. This Research Topic includes four papers addressing these issues.

Two are original research papers, one discusses a WBV protocol, and one is a literature review. The original papers (Tamini et al. and Pyatin and Maslova) discuss how WBV, administered during a three-week, in-hospital multidisciplinary body weight reduction program, increases resting energy expenditure (REE) in obese adolescents; and how the endocrine pathway modulates the osteogenesis and the immune system in postmenopausal women, eliminating osteoporosis during WBV. The protocol paper (Nantakool et al.) is about the effect of WBV training on functional capacity, vascular function, and glycemic control in individuals with type 2 diabetes (T2DM) and peripheral arterial disease (PAD). The review paper (Lu and Duan) discusses advances in WBV for treating osteoporosis.

For instance, obesity is associated with various clinical conditions, including endocrine system disturbances, and leads to multiple adverse health outcomes. Therefore, developing effective strategies to mitigate these consequences is essential (9), especially for children. Pediatric obesity is a growing global health concern, and interventions that increase REE have gained attention as a complementary strategy to dietary restriction. WBV therapy may offer metabolic and functional benefits, particularly in populations with limited exercise tolerance. Osteoporosis is a systemic skeletal disease characterized by reduced BMD and degeneration of the bone microstructure. It is a serious clinical condition in postmenopausal women and is strongly associated with disturbances in the endocrine system, particularly estrogen deficiency. Given this, developing effective osteoporosis management strategies is highly desirable (10). Current osteoporosis treatments face challenges such as drug side effects, low patient adherence, and comorbidities. As a non-invasive physical treatment, VT regulates bone metabolism through mechanical stress stimulation. It is emerging as an important complementary strategy in the comprehensive management of osteoporosis. Lu and Duan reviewed VT’s mechanisms of action and clinical efficacy in managing osteoporosis.

## Conclusion

As the interest in the biological effects of VT increases worldwide, it is worthwhile considering how the endocrine system responds to this therapy, which involves transmitting mechanical vibration to the human body. The presence of mechanoreceptors in the body supports this idea. This Research Topic explores the effects of VT on diseases related to the endocrine system, such as osteoporosis, obesity, and diabetes.
